# Evaluation of AI-based detection of incidental pulmonary emboli in cardiac CT angiography scans

**DOI:** 10.1007/s10554-025-03456-0

**Published:** 2025-07-07

**Authors:** Dana Brin, Efrat K. Gilat, Daniel Raskin, Orly Goitein

**Affiliations:** 1 Department of Diagnostic Imaging, Chaim Sheba Medical Center, Sheba 2, Tel Hashomer, 5266202 Israel; 2 Faculty of Medicine, Tel-Aviv University, Tel Aviv, Israel

**Keywords:** Cardiac CT angiography (CCTA), Pulmonary embolism, Artificial intelligence, Incidental findings

## Abstract

Incidental pulmonary embolism (PE) is detected in 1% of cardiac CT angiography (CCTA) scans, despite the targeted aortic opacification and limited field of view. While artificial intelligence (AI) algorithms have proven effective in detecting PE in CT pulmonary angiography (CTPA), their use in CCTA remains unexplored. This study aimed to evaluate the feasibility of an AI algorithm for detecting incidental PE in CCTA scans. A dedicated AI algorithm was retrospectively applied to CCTA scans to detect PE. Radiology reports were reviewed using a natural language processing (NLP) tool to detect mentions of PE. Discrepancies between the AI and radiology reports triggered a blinded review by a cardiothoracic radiologist. All scans identified as positive for PE were thoroughly assessed for radiographic features, including the location of emboli and right ventricular (RV) strain. The performance of the AI algorithm for PE detection was compared to the original radiology report. Between 2021 and 2023, 1534 CCTA scans were analyzed. The AI algorithm identified 27 positive PE scans, with a subsequent review confirming PE in 22/27 cases. Of these, 10 (45.5%) were missed in the initial radiology report, all involving segmental or subsegmental arteries (*P* < 0.05) with no evidence of RV strain. This study demonstrates the feasibility of using an AI algorithm to detect incidental PE in CCTA scans. A notable radiology report miss rate (45.5%) of segmental and subsegmental emboli was documented. While these findings emphasize the potential value of AI for PE detection in the daily radiology workflow, further research is needed to fully determine its clinical impact.

## Introduction

Pulmonary embolism (PE) is reported as an incidental finding in cardiac CT angiography (CCTA) in 1% [1–3]. The prevalence of PE in CCTA is higher in specific subgroups, reported in up to 6.2% following coronary artery bypass grafting (CABG) [4, 5] and in 7.9% of patients with acute stroke [6]. Since incidental and symptomatic PE have similar prognosis, the diagnosis of incidental PE is crucial in order to initiate the necessary treatment [2].

PE is not uncommonly missed, with reported miss rates ranging from 8 to 50% [7–10]. To our knowledge, there is no specific data on PE miss rate in CCTA. CCTA may be prone to misdiagnosis of PE due to technical factors such as the use of a small field of view (FOV) centered on the heart and optimal opacification of the aorta and coronary arteries instead of the pulmonary arteries.

In recent years, the integration of artificial intelligence (AI) into practiced medicine has increased, particularly in radiology, enhancing the routine workflow and patient care [11, 12]. Previous studies have documented the efficacy of AI algorithms in PE detection [8–10, 13–17]. These studies relied on CT pulmonary angiography (CTPA) scans or venous-phase contrast-enhanced chest CT scans for the evaluation of AI performance. However, the routine implementation of AI algorithms for PE detection in CCTA is yet to be reported.

This study aimed to compare the original radiology report and the performance of the AI algorithm for PE detection in CCTA scans.

## Methods

### CCTA data

We conducted a retrospective review of all CCTA studies from a single medical center that were acquired during a two-year period, between February 2021 and February 2023. These included coronary CTA, scans performed prior to transcatheter aortic valve implantation (TAVI), following CABG procedures, and scans for assessment of left atrial appendage (LAA) or prosthetic valve function.

The relevant scans were performed according to accepted protocols [18]. CCTA scans were acquired with ECG gating implementing dedicated protocols according to the relevant clinical question (CT scanner: GE Revolution, GE HealthCare, Chicago, Illinois, USA). Arterial enhancement was achieved using the bolus tracking technique with a region of interest in the descending aorta (for coronary, CABG, TAVI, and prosthetic valve protocols) or in the left atrium (LAA protocol). Contrast medium (60–70 mL Iomeron or Omnipaque 350/400) was injected at a rate of 4.5–5.5 mL/sec followed by a 50 mL chaser using a power injector (Nemoto, GE HealthCare). The scanning parameters were as follows: collimation of 160 mm (256 × 0.625 mm), axial scanning mode, with tube current adjusted according to the patient’s body mass index. The scan duration ranged from 0.3 to 0.5 seconds. Images were reconstructed with a slice thickness of 0.625 mm and a slice increment of 0.625 mm to produce overlapping isotropic voxels.

All scans were reconstructed using multiple reconstruction filters, including standard soft-tissue, angiography window, and lung window kernels. The reconstruction FOV was selected to primarily include the heart for most protocols (coronary CTA, CABG, LAA and prosthetic valve function), except for TAVI studies, which included a full thoracic FOV.

All the relevant radiology notes were reported by an attending cardiothoracic radiologist, without the assistance of an AI algorithm.

### AI algorithm

A dedicated AI algorithm (Aidoc Medical, Tel Aviv, Israel) was utilized to retrospectively analyze all CCTA scans for the presence of PE. This is a commercially available AI-based image analysis software defined as a radiology support tool. The algorithm is designed to detect incidental PE across various contrast-enhanced CT protocols, irrespective of pulmonary artery enhancement levels [19]. The algorithm was trained on data primarily involving a full thoracic FOV, but not exclusively; thus, it is also applicable to scans with a limited FOV. This algorithm has been previously validated for incidental PE detection in contrast-enhanced chest CT across multiple studies [10, 20, 21].

### Reference standard

The original radiology reports were screened for the presence of PE using a natural language processing (NLP) system, provided by Aidoc Medical. This NLP system was validated in a previous multi-institutional study with a sensitivity of 99.1% and specificity of 96.4% for classifying radiology reports as positive or negative for PE [22]. Scans with discrepancies between the radiology report and the AI software regarding the presence of PE were reviewed in consensus by an experienced cardiothoracic radiologist with 20 years of experience (OG) and a second-year radiology resident (DB), who were blinded to both readings. Scans identified as positive for PE by both the radiology reports and the AI algorithm were reviewed manually for confirmation and further analysis. Scans that were categorized as negative by both the radiology report and the AI software were not further reviewed. This method is in line with previous studies that have utilized NLP for extracting information regarding PE presence from radiology reports [10, 20]. Nonetheless, it may underestimate the false negatives due to the reliance on the NLP for review of negative cases.

### AI detection results

The performance of the AI software for PE detection was retrospectively evaluated. The AI results were classified as true positive (TP), false positive (FP), true negative (TN) and false negative (FN). Sensitivity, specificity, negative predictive value (NPV), positive predictive value (PPV), accuracy, F1 score, MCC and precision were calculated.

### PE radiographic features

**Fig. 1:**
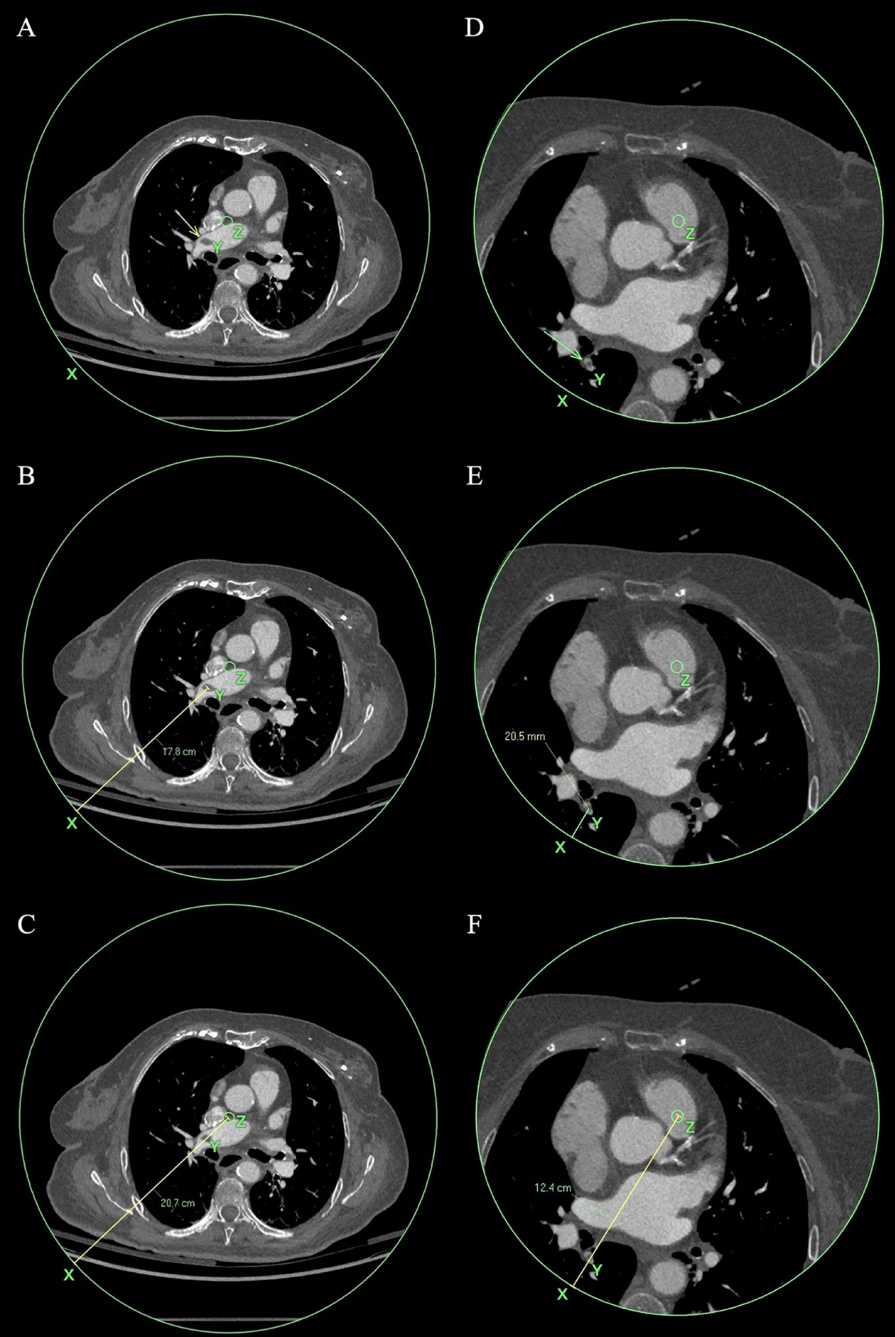
Measurement of Periphery Index in two cardiac CT angiography cases: Case 1: Transcatheter aortic valve implantation (TAVI) protocol in an 82-year-old female with pulmonary embolism (PE) in the right main pulmonary artery (images A-C). Case 2: Left atrial appendage (LAA) protocol in a 76-year-old female with subsegmental PE in the right lower lobe (images D-F). This protocol has a limited field of view (FOV), focusing on the heart. In each case: X marks the edge of the FOV, shown as a large green circle, Y marks the location of the PE (arrows in images A and D), Z marks the center of the FOV, shown as a small green circle. The Periphery Index is calculated as the ratio between the distance from the PE to the edge of the image (XY) and the radius of the FOV (XZ). Case 1 (images A-C): XY = 17.8 cm (image B), XZ = 20.7 cm (image C) -> Periphery index = 0.86. Case 2 (images D-F): XY = 20.5 mm = 2.05 cm (image E), XZ = 12.4 cm (image F) -> Periphery index = 0.17. A higher Periphery Index (closer to 1.0) reflects a more central PE in the image, while a lower index indicates a more peripheral location

Each positive PE scan (either by the radiology report or by AI detection) was assessed for several radiographic features. The level of PE was defined as main pulmonary artery (PA), lobar, segmental or subsegmental, according to the most proximal level of PE. For the purpose of this study, we defined the term “periphery index” calculated as the ratio between the distance of the most proximal PE from the periphery of the image and the radius of the FOV (Fig. 1). This index indicates the peripheral or central location of each PE in the specific scan. PA vascular enhancement quality was evaluated by comparing the contrast enhancement of the aorta and main PA. Vascular enhancement, measured in Hounsfield units (HU), of the ascending aorta and main PA was obtained at the level of the main PA bifurcation (on the same slice). The aorta/PA enhancement ratio (A/P ratio) was therefore calculated. Right ventricular (RV) strain was documented as the ratio between the maximal RV and the left ventricle (LV) diameters (RV/LV ratio). These imaging features were compared between PE cases that were reported in the original radiology report and PE cases that were originally missed by the interpreting radiologist.

### Statistical analysis

The statistical analysis was performed using Python version 3.11.8. Statistical significance was determined using a p-value threshold of less than 0.05. T-test and Mann-Whitney U test were employed to assess differences between reported and unreported PE cases. T-test was used for comparing PA enhancement, distance from image center, distance from the edge of the field of view, periphery index, and RV/LV ratio. Mann-Whitney U test was used to compare A/P ratio and level of PE.

An Institutional Review Board (IRB) approval was granted for this study. The IRB committee waived informed consent, due to the retrospective nature of this study.

## Results

### CCTA scans data

In the period between February 2021 and February 2023, 1534 CCTA scans were performed in a single medical center. CCTA scans’ indications are presented in Table 1.

**Table 1:** Cardiac CT angiography scans acquired during a two-year period, according to CT protocol

Cardiac CTA protocol	Number (%)
TAVI	669 (43.6)
Coronary CTA	560 (36.5)
CABG	40 (2.6)
LAA	258 (16.8)
Prosthetic valve function	7 (0.5)
Total	1534 (100)

Abbreviations: Computed tomography angiography (CTA), transcatheter aortic valve implantation (TAVI), coronary artery bypass grafting (CABG), left atrial appendage (LAA)

### Presence of PE

**Fig. 2:**
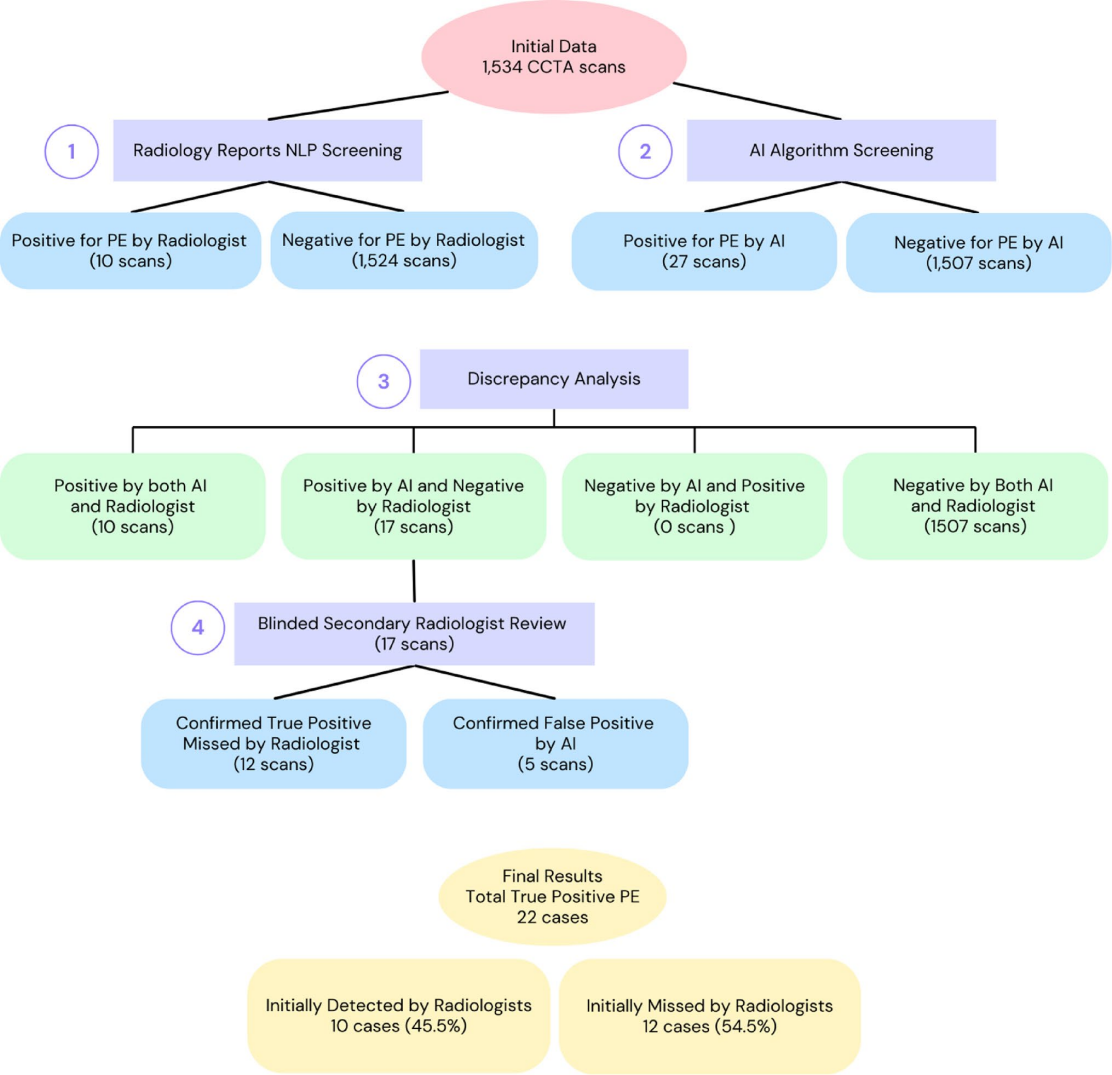
Retrospective analysis flowchart

The AI algorithm identified 27/1534 CCTA scans as positive for PE (1.8%). The NLP algorithm found 10 radiology reports that mentioned PE presence. All cases recognized by the NLP algorithm were also read as positive by the AI algorithm. Discrepancies between the AI algorithm and the radiology reports, analyzed by the NLP, were found in 17 scans, which underwent a repeated reading of both scan and report. This confirmed PE presence in 12/17 cases and refuted this diagnosis in the remaining five cases. Thus, the final true positive cases (identified by both the AI algorithm and the radiologist in the repeated reading) were 22/27 and the false positives by the AI algorithm reading were 5/27. Of the 22 true positive scans, 10 reports were identified as positive for PE by the NLP algorithm and two additional cases were missed (identified in the second reading of the report, i.e., false negative by the NLP algorithm). The initial radiology interpretation missed PE presence in 10/22 true positive PE cases (45.5% miss rate) (Fig. 2).

### Performance of the AI algorithm

In comparison with the primary radiology report analyzed by NLP, the AI algorithm performance had 100% sensitivity, 99.6% specificity, 99.6% accuracy, 81.4% PPV, and 100% NPV. The AI algorithm demonstrated an F1 score of 89.8%, an MCC of 90%, and a precision of 81.4%.

### Radiographic features of PE cases

**Fig. 3:**
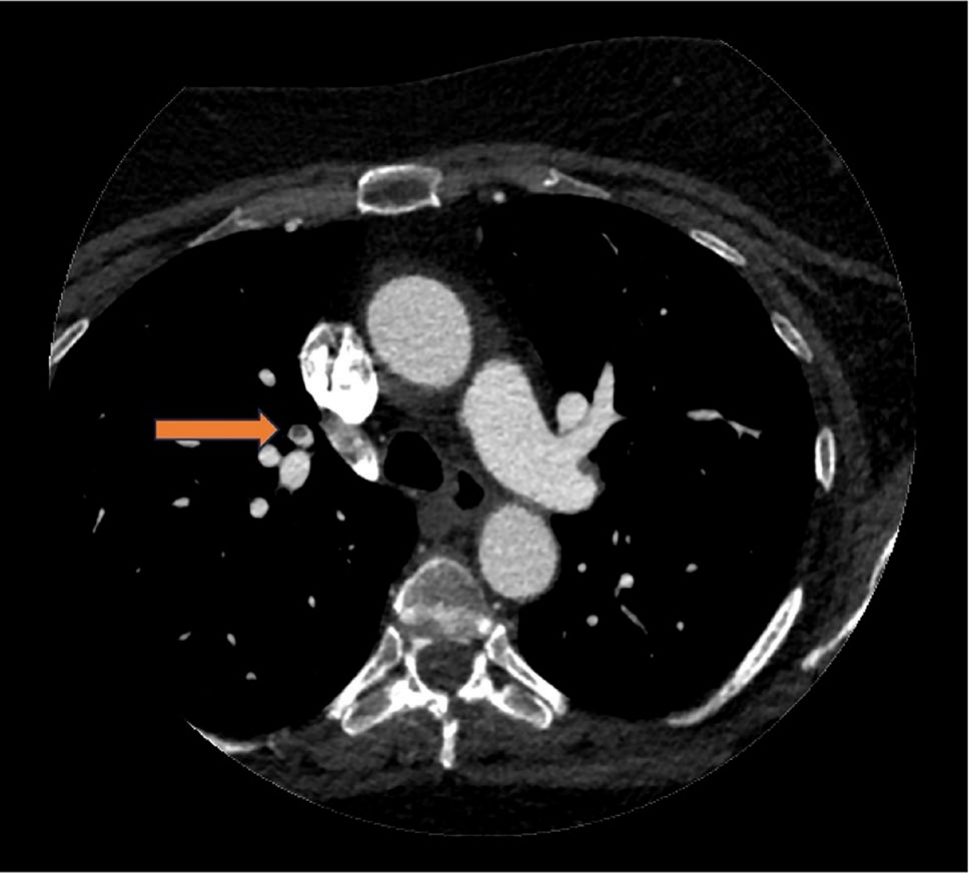
Right upper lobe segmental pulmonary embolism (PE) identified in a coronary artery CTA from an asymptomatic 60-year-old female with cardiovascular risk factors. Initially unreported in the original radiology report, this PE was later detected by the AI algorithm

**Fig. 4:**
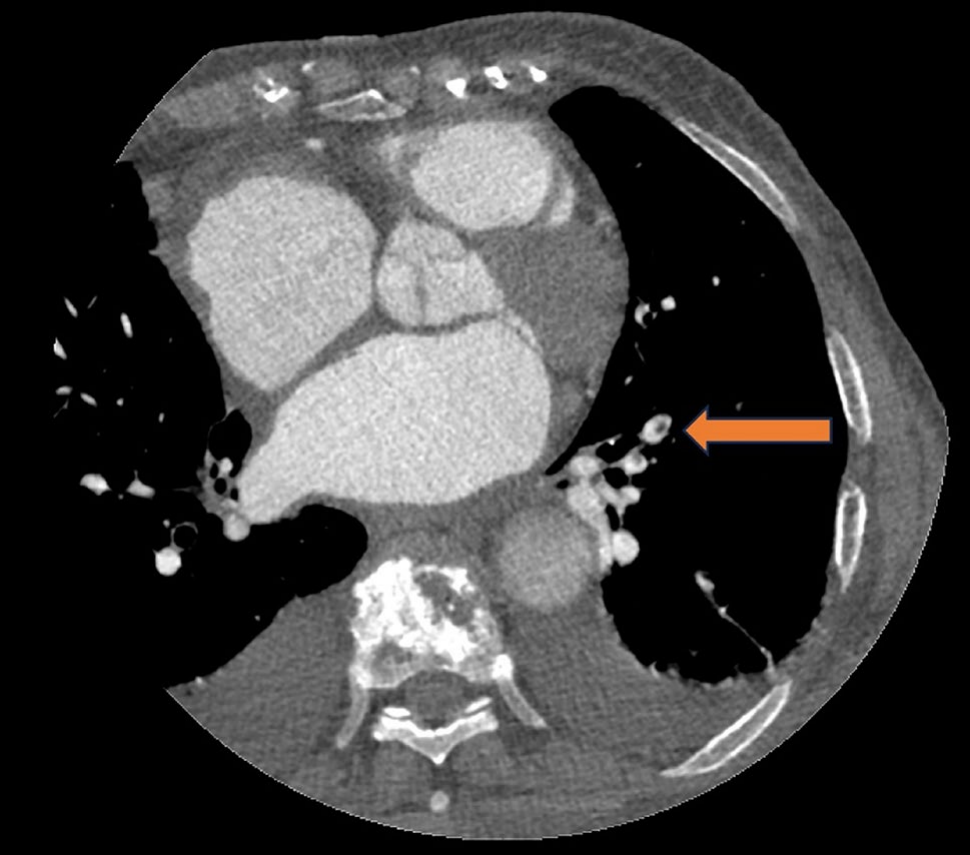
Left lower lobe subsegmental pulmonary embolism (PE) on a CTA targeting the left atrial appendage of an 88-year-old female patient. The PE was not reported in the initial radiology report, but was subsequently identified by the AI algorithm

The radiographic features of all PE positive cases are summarized in Table 2. The only statistically significant difference between the reported and unreported PE cases was the level of PE. All unreported cases were detected in segmental or subsegmental arteries (*P* < 0.05) (Figs. 3 and 4). There was no evidence of RV strain in any of the positive PE cases, as reflected by a mean RV/LV ratio of up to 1.1. Comparing the reported and unreported PE cases, there was no statistically significant difference regarding the A/P ratio, PA enhancement, periphery index, and distances from the center or edge of the image (Table 2).

**Table 2:** Radiographic features of CCTA scans positive for PE, in reported and unreported PE cases

	All positive incidental PE cases	PE cases unreported in the original radiology report	PE cases reported in the original radiology report	*P* value
PA enhancement (mean HU)	428.6	425.4	431.3	0.9
A/P ratio^a^ (mean)	1.2	0.9	1.4	0.428
Level^b^ (n) Main Lobar Segmental Subsegmental	3 4 7 8	0 0 3 7	3 4 4 1	0.001
Distance from the center of the image (mean cm)^c^	6.4	7	6	0.332
Distance from the edge of the image field of view (mean cm)^c^	6.3	6.3	6.3	0.99
Periphery index (mean)^d^	0.5	0.5	0.5	0.81
RV/LV ratio (mean)	1	1.1	0.9	0.182

^a^A/P ratio calculated as the ratio between the contrast enhancement in Hounsfield units of the ascending aorta and the pulmonary trunk, measured at the level of the pulmonary trunk bifurcation

^b^Level of the PE is defined according to the highest level of PE found in the scan

^c^Measured in cm for the most proximal PE finding in each scan

^d^Calculated as the ratio between the distance from the edge of image field of view and the radius of the field of view. A higher periphery index means the PE finding is central

Abbreviations: Cardiac computed tomography angiography (CCTA), Pulmonary emboli (PE), Pulmonary artery (PA), Hounsfield units (HU), Right ventricle (RV), Left ventricle (LV)

## Discussion

This study evaluates the effectiveness of an AI algorithm in detecting incidental PE in CCTA scans. To our knowledge, this specific application has not been addressed in the literature. In total, there were 22 cases positive for PE, out of which 10 (45.5%) were missed by the original radiology report. The only statistically significant difference between the reported and unreported PE cases was the level of PE. All missed PE cases were segmental or subsegmental with no evidence of RV strain.

The clinical significance of subsegmental PE is controversial [23–25]. Graeve et al. reported that AI-enhanced detection of subsegmental PE did not significantly affect short-term patient survival [7]. In contrast, Le Gal et al. found that patients with subsegmental PE who were not treated with anticoagulation, had a higher-than-expected rate of recurrent venous thromboembolism over a 90-day follow-up [26]. These findings suggest that subsegmental PEs cannot be disregarded and underscore the need for further research to determine optimal management strategies.

The AI algorithm demonstrated high sensitivity (100%), specificity (99.6%) and accuracy (99.6%) in accordance with its performance in CTPA scans as reported in prior research. The reported sensitivity and NPV of the AI algorithm should be interpreted with caution, as the study design did not include a complete evaluation of negative cases, potentially inflating these metrics. Analysis of AI algorithm efficacy in CTPA scans documented a sensitivity and specificity range of 73-96.8% and 95-99.9% respectively, with pooled values from a meta-analysis of 88% and 86% [8, 9, 14, 16, 27].

The current study documented incidental PE in 1.4% of CCTA scans, consistent with the existing literature [1–3]. Yet, we documented a 45.5% radiology report miss rate for PE diagnosis in CCTA scans. This is in discordance with the reported miss rate of 8.4-10.8% in CTPA scans [8, 9] and similar to the 37.3% miss rate in portal venous phase chest CT scans [10]. An inherent difference between dedicated CTPA and CCTA protocols is the PA enhancement level. CTPA scans are targeted for optimal PA opacification whereas CCTA scans aim to obtain the best aortic and coronary enhancement. In our study, although the PA attenuation was sufficient with a mean value of 428.6 HU and a slightly higher attenuation in the reported PE cases compared to unreported PE cases, this difference was not statistically significant.

The radiologist’s interpretation of a scan is influenced by the clinical information presented, a phenomenon known as “framing bias” [28]. A noteworthy difference between the CTPA and CCTA protocols is the specific aim of the study and the main clinical question. CTPA is performed to assure or rule out the presence of PE. On the other hand, CCTA scans are performed for a myriad of clinical indications, including evaluation of the coronary arteries, post-CABG, LAA thrombi, and prior to TAVI procedures. None of these indications include specific or targeted evaluation of the pulmonary arterial tree, contributing to potential “inattentional blindness” among radiologists [29]. Radiologists are subject to additional biases that contribute to errors in diagnosis. “Satisfaction of search” bias refers to decreased awareness of additional abnormalities after the first pathology is identified [28]. These biases could explain in part why radiologists tend to miss more PE cases when not directly asked about PE presence and when other pathologies are found in the scan.

Another caveat of CCTA is the limited FOV. Previous research showed that the use of a limited FOV in CCTA scans resulted in missing nearly 90% of lung cancers detected by full thoracic scanning [30]. Similarly, two-thirds of lung nodules were missed in CCTA scans with limited FOV [31]. Previous studies found that most of the unreported PE cases in CTPA scans were located in segmental and subsegmental arteries [8]. Radiologists’ CTPA PE detection rate was documented as being higher for central emboli in comparison to subsegmental PE [16]. A recent study assessing PE in cancer patients, found that unreported cases had a significantly lower number of involved vessels compared to reported cases [21]. Similarly, in our study all unreported PE scans were documented either in a segmental or subsegmental location. Another factor affecting errors in diagnosis is the continuous increase in scan volume, workload and burnout amongst interpreting radiologists [18].

Comparisons between the performance of the AI algorithm and the interpreting radiologist in PE diagnosis have yielded mixed results [8–10], with some studies suggesting superior AI accuracy. In addition, some AI platforms are able to quantify PE severity, further stressing the diagnostic benefits of its incorporation into the daily workload [32]. Therefore, the combination of the lack of a specific clinical indication to rule out PE, the sub-optimal opacification of the pulmonary tree, the limited FOV, the low prevalence of PE (1.4% in our study), the segmental or subsegmental location, and the increased work overload, result in a documented higher miss rate of PE by the interpreting radiologist. Our findings suggest that the routine integration of AI for PE detection in CCTA scans is of major importance. Thus, AI implementation might provide support for the interpreting radiologist in this setting.

The limitations of this study include a small number of positive PE cases and the potential underestimation of false negatives due to the reliance on NLP for initial report reviews and the lack of a comprehensive reference standard. Additionally, the retrospective design and selective review of discordant cases may have led to an overestimation of the AI algorithm’s sensitivity and negative predictive value. These limitations highlight the importance of larger, prospective studies with thorough reference standards to validate the performance of AI algorithms in detecting incidental PE in CCTA scans.

In conclusion, the study highlights the feasibility of using AI for detecting incidental PE in CCTA scans. The results suggest that AI could serve as a valuable adjunct to radiologists, particularly for identifying segmental and subsegmental PE. Thus, further research is needed to determine its clinical impact and optimal integration into routine workflows.

## Data Availability

No datasets were generated or analysed during the current study.
